# Mechanism of Action and Interactions between Thyroid Peroxidase and Lipoxygenase Inhibitors Derived from Plant Sources

**DOI:** 10.3390/biom9110663

**Published:** 2019-10-29

**Authors:** Ewa Habza-Kowalska, Urszula Gawlik-Dziki, Dariusz Dziki

**Affiliations:** 1 Department of Biochemistry and Food Chemistry, University of Life Sciences, Skromna Str. 8, 20-704 Lublin, Poland; ewa.habza1@gmail.com; 2 Department of Thermal Technology and Food Process Engineering, University of Life Sciences, Głęboka 31, 20-612 Lublin, Poland; dariusz.dziki@up.lublin.pl

**Keywords:** thyroid peroxidase, lipoxygenase, inhibition, dietary polyphenols, antioxidant activity

## Abstract

This study focused on the effect of kaempferol, catechin, apigenin, sinapinic acid, and extracts from plants (i.e., parsley, cumin, mustard, green tea, and green coffee) on thyroid peroxidase (TPO) and lipoxygenase (LOX) activity, antiradical potential, as well as the result of interactions among them. Catechin, sinapinic acid, and kaempferol acted as a competitive TPO inhibitors, while apigenin demonstrated an uncompetitive mode of inhibitory action. Ethanol extracts from all plants acted as competitive TPO inhibitors, while, after in vitro digestion, TPO activation was found especially in the case of mustard (24%) and cumin (19.85%). Most importantly, TPO activators acted synergistically. The TPO effectors acted as LOX inhibitors. The most effective were potentially bioaccessible compounds from green tea and green coffee (IC_50_ = 29.73 mg DW/mL and 30.43 mg DW/mL, respectively). The highest free radical scavenging ability was determined for catechin and sinapinic acid (IC_50_ = 78.37 µg/mL and 84.33 µg/mL, respectively) and potentially bioaccessible compounds from mustard (0.42 mg DW/mL) and green coffee (0.87 mg DW/mL). Green coffee, green tea, cumin, and mustard contain potentially bioaccessible TPO activators that also act as effective LOX inhibitors, which indicate their potentially health-promoting effects for people suffering from Hashimoto’s disease.

## 1. Introduction

Thyroid peroxidase, also called thyroperoxidase (TPO, EC 1.11.1.1-14) or iodide peroxidase, catalyzes iodide oxidation to form iodine atoms which are added onto tyrosine residues on thyroglobulin for the production of thyroxine or triiodothyronine, i.e., thyroid hormones [1]. Thyroid peroxidase is the major antigen in human Hashimoto’s disease, and anti-TPO antibodies induce complement-dependent cytotoxicity. Furthermore, antibodies against complement (anti-C1q) are detected in patients with Hashimoto’s disease. They are correlated with thyroid-stimulating hormone (TSH) levels [2]. During common thyroid disorders (hyper- and hypothyroidism) the activity level of TPO is changed. Thyroiditis is the inflammation of the thyroid gland due to the numerous etiologies [3]. Graves’ disease accounts for 50–80% of cases of hyperthyroidism. The most commonly used group of anti-thyroid drugs in patients with Graves’ disease are thionamides: methimazole (MMI), propylthiouracil (PTU), and carbimazole (CBZ). Their main effect is to inhibit the synthesis of thyroid hormone by blocking the action of TPO [4].

Hashimoto’s thyroiditis (HT) (i.e., chronic lymphocytic thyroiditis) is an autoimmune disease that causes the immune system to attack and destroy the thyroid gland. The resulting inflammation often leads to an underactive thyroid gland (i.e., hypothyroidism). The disease affects between 0.1% and 5% of all adults in Western countries. Hashimoto’s thyroiditis has been associated with thyroid carcinoma and malignant lymphoma of the thyroid. In addition, patients with HT were at higher risk for breast and lung cancer than those in the control group. However, a number of studies did not confirm such an association with thyroid, breast, and lung cancers [5]

Inflammation is a natural defense mechanism against pathogens and is associated with many pathogenic diseases. Many of them are linked with higher production of reactive oxygen species (ROS) resulting in oxidative stress and a variety of protein oxidation events. One of the main enzymes whose activity induces oxidative stress are lipoxygenases (LOXs) [6]. In animal and cell culture studies, activation of the lipid-oxidizing enzyme 12/15-lipoxygenase (12/15-LOX) plays a central role as an inflammatory mediator in the pathology of oxidative stress. The mechanism of 12/15-LOX involves the production of reactive oxygen species through the metabolism of arachidonic acid as well as direct detrimental effects on organelle membranes [7].

Lipoxygenases (LOXs, EC. 1.13.11.12) are widely known for their presence in plants and animals. They are known as nonheme iron-containing dioxygenases. Lipoxygenase isozymes take part in the metabolism of eicosanoids, e.g. prostaglandins, or non-classic eicosanoids [8]. Human lipoxygenases are located on chromosome *17.p13* with the exception of the *5*-LOX gene (located on chromosome *10q11.2*) [5]. The described human lipoxygenases along with their products are associated with such conditions as inflammatory and allergic diseases, atherosclerosis, and several types of cancers [9]. Lipoxygenase is an enzyme with activity that is related with oxidative stress in the human body. The activity of ROS influence the cells’ death.

Under normal physiological conditions, the thyroid gland participates in the autoregulation of the redox balance. Excessive ROS activity can disturb this balance, which can affect thyroid enzyme activity. This mechanism is still unclear and some studies should be undertaken to explore this occurrence [10]. Inflammation and oxidative stress (OS) are closely related processes. Among the various hormonal influences that operate on the antioxidant balance, thyroid hormones play particularly important roles, since both hyperthyroidism and hypothyroidism have been shown to be associated with OS in animals and humans [11].

It is known that plants are a rich source of secondary metabolites (i.e., antioxidants) such as polyphenols with documented biological activity. Many polyphenols are known as bioactive substances with antioxidative, antimutagenic, antibacterial, and antiviral activity. To exhibit their biological properties, polyphenols have to be available to some extent in the target tissue. Therefore, the biological properties of dietary polyphenols may depend on their absorption in the gut and bioavailability. The amount of bioaccessible food-related polyphenols may differ quantitatively and qualitatively from polyphenols included in food databases. Moreover, most studies on polyphenol bioavailability used mainly pure single molecules (isolated from food or chemically synthesized), although their bioavailability from whole foods may be substantially different [12]. Furthermore, the activity of phenolic compounds studied in vitro (after isolation thereof from food) does not have to coincide with the activity demonstrated in the human organism. In vitro models based on human physiology are simple, cheap, and repeatable tools for studying the bioaccessibility of food components. They are widely used to investigate structural changes, digestibility, and release of food components in simulated conditions of the alimentary tract [13].

In contrast to synthetic pharmaceuticals, based upon single chemicals, many phytomedicines exert their beneficial effects through the additive or synergistic action of several chemical compounds acting at single or multiple target sites associated with a physiological process [14]. However, the synergism of bioactive compounds plays a considerable role; however, there are only a few studies of this issue in such a complicated system as whole foods.

The method used for identification of the interactions among active compounds is referred to as isobolographic analysis. isobolographic analysis is a useful tool for determination of the interactions among components of two-component mixtures as well as those composed of plant extracts being mixtures of many active compounds [15].

This study focused on the influence of biological active substances contained in food components on TPO and LOX activity, as well as the impact of interactions among active substances contained in plant food sources on TPO activity. The results were compared with those obtained for pure chemical compounds which helped draw conclusions about the impact of the food matrix on the biological activity of phytochemicals. The present study determined the effect of four frequently occurring dietary polyphenols (kaempferol, catechin, apigenin, and sinapinic acid) and extracts from five plants rich in such polyphenols (i.e., parsley, cumin, mustard, green tea, and green coffee) on TPO and LOX activity in order to explain these mechanisms. Polyphenols were chosen on the basis of the database of natural polyphenols [16] and preliminary studies of the influence of pure polyphenols and selection of those which simultaneously act as TPO and LOX inhibitors (data unpublished). Plants were selected based on a database of natural polyphenols [16].

## 2. Materials and Methods

### 2.1. Chemicals

Sucrose (α-D-glucopyranosyl-(1→4)-β-D-fructofuranoside), tris (1,3-propanediol-2-amino-2-hydroxymethyl), KCl, NaCl, MgCl2, 90% ethanol, NaOH, guaiacol (2-methoxyphenol), H_2_O_2_ (hydrogen peroxide), ABTS (2,2′-azinobis-(3-ethylbenzothiazoline-6-sulfonic acid), lipoxygenase (LOX), xanthine oxidase (XO), xanthine, pancreatin, pepsin, bile extract, linoleic acid, α-amylase, sinapinic acid, apigenin, catechin, and kaempferol were purchased from Sigma–Aldrich (Poznan, Poland). All other chemicals were of analytical grade.

### 2.2. Material

Porcine thyroid glands were obtained at a local slaughterhouse (Lubmeat S.A., Lublin, Poland) and stored at −20 °C until used. The experimental material consisted of lyophilized parsley leaves, green coffee, green tea, cumin, and mustard which were bought from a local supermarket (TESCO, Lublin, Poland).

### 2.3. Preparation of Pure Substance Solutions

Sinapinic acid, catechin, apigenin, and kaempferol were diluted in 50% ethanol to concentrations 0.125 µg/mL, 0.25 µg/mL, 0.5 µg/mL, 1.0 µg/mL, 2.0 µg/mL, 25 µg/mL, 50 µg/mL, 100 µg/mL, 200 µg/mL and used for further assay.

### 2.4. Raw Extracts Preparation

For extraction 1.5 g of individual vegetables were homogenized with 15 mL of 50% ethanol and, subsequently, the samples were shaken for 30 min at room temperature. After centrifuging (15 min, 4000 rpm), the extraction procedure was repeated. The final volume was brought to 50 mL with 50% ethanol. The final extract concentration was 30 mg/mL. Extracts were then diluted to a concentration 0.3 mg/mL and 3 mg/mL.

### 2.5. High-Performance Liquid Chromatography-Diode-Array Detector Analysis

The high-performance liquid chromatography-diode-array detector (HPLC-DAD) analysis was carried out on a Shimadzu Model SPD-M20A 230V equipped with a column (COSMOSIL 5Diol-20-II Packed Column 7.5 mm ID × 300 mm, UVISON TECHNOLOGIES LIMITED Wrotham, England), city, state (if U, country). For analysis, a 100 µL sample of the extract was injected onto a column. Acetic acid (1%) with formic acid (0.1%, *v*/*v*) and acetonitrile with 0.1% formic acid were used as mobile phases A and B, respectively. The column temperature was maintained at 30 °C. Polyphenols were monitored at 280 nm [17]. Pure phenolic peaks (apigenin, catechin, kaempferol, sinapinic acid) in four concentrations (25 µL, 50 µL, 100 µL, 200 µL) were used to prepare the standard curve. The number of phenolic compounds (µg/g dry weight) was calculated by comparison of the peak areas of the samples with those of standards.

### 2.6. In Vitro Digestion

In vitro digestion of plant materials was performed according to Minekus et al. [18] with some modifications. The amount of 0.5 g of plant source was mixed with 6 mL of simulated saliva fluid (SSF). 22.75 mL of SSF, 0.163 mL CaCl_2_, and 3.25 mL of amylase were added to sample probe. The mixture was incubated for 2 min at 37 °C. The gastric phase was performed as follows: 48.75 mL of simulated gastric fluid SGF, 4.518 mL of H_2_O, 1.3 mL of HCl, and 10.4 mL of pepsin were mixed with a sample probe after a simulated oral phase, brought to pH 2.0, and incubated for two hours. The intestinal phase was performed as described in the protocol. A blank sample was prepared with 0.5 mL of distilled water. In the end, the mixture was centrifuged at 4500 rpm for 15 min. The supernatant was then stored at -20 °C until further analysis.

### 2.7. In Vitro Antioxidant Capacity Assay

The ABTS radical scavenging activity was determined according to Re et al. [19], with some modifications. A 250 µL ABTS solution was mixed with 10 µL of each pure polyphenols solutions (concentration 50 µg/mL), ethanol extracts, and digested extracts (concentrations: 0.3 mg/mL), and then was measured at the wavelength 724 nm using a UV/Vis spectrophotometer (BioTek, Model Epoch2TC, Winooski, VT, USA) after 15 min of incubation at room temperature. The inhibition percentage of ABTS discoloration was calculated using the fallowing equation:

AA = [(Ac − Ap)/(Ac)] × 100%(1)

where Ac is the absorbance of control, Ap is the absorbance of pure polyphenols solutions

The half maximal inhibitory concentration IC_50_ value was determined by interpolation of the dose–response curves. The IC_50_ values were calculated at fitted models as, the concentration of the tested compound gave 50% of the maximum inhibition based on a dose-dependent mode of action.

### 2.8. Thyroid Peroxidase Preparation

The assay was prepared according to Jomaa et al. [20] with some modifications. Frozen thyroid gland was cut into slices and homogenized in buffer containing 0.25 M sucrose, 2 mM Tris-HCl, 100 mM KCl, 40 mM NaCl, and 10 mM MgCl2 (pH 7.4) with Philips homogenizer. The thyroid gland was than centrifuged two times at 4000 RPM for 15 min at 4 °C. The enzyme protein was then salted-out to 60%. The supernatant was used for further analysis and stored at −20 °C.

The activity of the enzyme was assayed using guaiacol assay. The reaction mixture contained: 33 mM guaiacol, 0.27 mM H_2_O_2_, and 33 mM prepared sucrose buffer. The reaction components were incubated in 37 °C before assignment. The absorbance was determined using a Shimadzu spectrophotometer (Model UV-1280, Shimadzu Corporation, Kyoto, Japan) at a wavelength 470 nm. The assay was conducted as follows: to a cuvette, 180 µL of buffer, 100 µL of guaiacol, and 40 µL of TPO were mixed to the final volume of 420 µL. The cuvette was then placed into the spectrophotometer and the reaction was started by the addition of 100 µL H_2_O_2_. Absorbance readings were recorded every minute for a total of 3 min. Verification of the TPO activity was accomplished by linearly correlating TPO concentration with absorbance readings.

### 2.9. Thyroid Peroxidase Inhibitory Assay

The assay was used according to Jomaa et al. [20] with some modification. The measurement was made using a plate spectrophotometer (BioTek) in 96 well plates at a wavelength of 470 nm. The assay was conducted as follows: 50 µL of buffer, 40 µL of pure substance solution or ethanol extracts solution or in vitro digested solution, 50 µL of guaiacol, 20 µL of TPO enzyme, and 50 µL H_2_O_2_. The total volume of the mixture was 210 µL. In the sample probe, extracts were replaced by buffer. Absorbance readings were recorded every minute for a total of 3 min at 37 °C, as a unit of TPO activity is defined as the change in the absorbance per minute. All measurements were performed in three replicates.

The TPO inhibitory activity was calculated as follows:

%inhibition = (1− (∆A/〖min〗_test )/(∆A〖min〗_blank )) × 100%(2)

where ΔA/min test is the linear absorbance change per minute of the test material and ΔA min blank is the linear change in absorbance per minute of blank.

The IC_50_ value was determined by interpolation of dose–response curves. The IC_50_ values were calculated at fitted models as the concentration of the tested compound that gave 50% of the maximum inhibition based on a dose-dependent mode of action. The mode of inhibition of the enzyme was performed using the Lineweaver–Burk plot.

### 2.10. Inhibition of Lipoxygenase Activity

The inhibition of LOX with linoleic acid as a substrate was measured spectrophotometrically, based on Axelrod et al. [21] and adopted for a microplate reader (Epoch 2 Microplate Spectrophotometer, BioTek Instruments). The mixture contained 240 µL 0.066 M phosphate buffer, 10 µL LOX solution, 10 µL pure substance solution, ethanol extract solution or digested extract. The reaction was started by adding 40 µL 2.5 mmol/L linoleic acid. For a unit of LOX activity, the change in the absorbance per minute at the wavelength 234 nm was defined. All measurements were performed in three replicates.

The IC_50_ values were calculated at fitted models as the concentration of the tested compound that gave 50% of the maximum inhibition based on a dose-dependent mode of action. The mode of inhibition of the enzyme was performed using the Lineweaver–Burk plots.

### 2.11. Isobolographic Analysis

The results (type of interactions) can be shown on isobolograms as per Chou’s method [15]. Synergism means that two components mutually enhance their activities (concave isobole) antagonism means that two components decrease the effect of the single component (convex isobole), and additive interaction (straight line) [15].

For this assay, only substances with a 100 µg/mL concentration were used (according to previous results). Pure substances were mixed in various volume ratio: 3:2, 2:3, and 1:1. All of mixtures were made in combinations of two pure substances or two extracts (digested in concentrations of 0.3 mg/mL).

### 2.12. Statistical Analysis

All tests were performed in triplicate. The results were statistically analyzed in the Statistica. One-way analysis of variance (ANOVA) was made with the significance level α = 0.05. Tukey’s test was used to evaluate the differences among means.

## 3. Results and Discussion

### 3.1. High-Performance Liquid Chromatography-Diode-Array Detector Phenolic Analysis

As previously mentioned, fruits and vegetables are rich sources of polyphenols. In present study, five plant extracts were used as a source of different polyphenols. Green tea and green coffee are a rich source of catechin; however, a significantly higher content was found in green coffee beans (Table 1). Apigenin can be found in parsley leaves, kaempferol in cumin, and sinapinic acid in mustard. The content of each polyphenol is shown in Table 1.

The data concerning kaempferol content in cumin were in accordance with those provided by Shan et al. [22]. Studies conducted by Ani et al. [23] showed a higher amount of kaempferol (94.70 µg/g DW) detected in *Cumin nigrum* seeds.

The content of apigenin in parsley depends on the place where it was bought. Such conclusions were drawn by Głowacki et al. [24]. In our study, the lower content of apigenin was determined (0.69 mg/g DW). On the other hand, it was higher than that given by Yashin et al. [25]. The concentration of sinapinic acid in mustard detected by Engels et al. [26] (2.66 mg/g) was higher than that obtained in our study; however, no alkaline hydrolysis was carried out in our studies. The content of catechin depends on the source of the tea or coffee. Our results are in agreement with those obtained by Henning at al. [27]. Of course, plant extracts contain the entire spectrum of polyphenols, and the activity depends on their composition and interactions. Stan et al. [28] identified flavones apigenin and luteolin and the flavonols quercetin and kaempferol in ethanolic extracts from parsley leaves using an HPLC Shimadzu apparatus equipped with PDA and MS detectors. In mustard cotyledons and hulls, the major phenolics were sinapine (SP), with small amounts of sinapoyl glucose (SG), and sinapinic acid (SA) with a significant difference in phenolic contents among the two seed fractions. Cotyledons showed a relatively high content of SP, SA, SG, and total phenolics in comparison to hulls [29]. In the study by Acimovic at al. [30], hydroxybenzoic and hydroxycinnamic acids, as well as glycosides of flavonones and flavonoles were most abundant in the cumin samples from Serbia. The phenolic content of green tea is widely diverse, although catechins are the major constituents; however, other flavonoids and phenolic acids have also been identified [31].

Thus, an additional goal of our work was to show that predicting the activity of plant extracts based only on the content of one specific compound (as it happens in the standardization of plant extracts) can lead to errors.

### 3.2. Antioxidant Analysis

The antioxidant capacity of pure polyphenols (i.e., kaempferol, apigenin, catechin, and sinapinic acid), ethanolic, and digested extracts was evaluated by the most commonly used antioxidant assay—the ABTS method. The ABTS radical scavenging abilities of catechin and sinapinic acid were found to be the highest (78.37 µg/mL and 84.33 µg/mL, respectively). The lowest antiradical activity was observed for apigenin (405.93 µg/mL) (Table 2).

The present results show the mutual dependence between pure polyphenolic solutions (Table 2) and plant extracts, which are a source of the investigated polyphenols (Table 1). As expected, the antioxidant activities of plant extracts were significantly lower than the activity of the pure compounds; however, the antiradical activity of the tested extracts was relatively high. The lowest antiradical activity (IC_50_ = 3.47 mg DW/mL) was determined for parsley ethanolic extract. Hinneburg et al. [32] reported that the parsley hydro-distilled extract showed an IC_50_ value of 12.0 ± 0.10 mg/mL in the DPPH scavenging assay. This shows that phenolic compounds of parsley could be responsible for the observed DPPH radical scavenging activity, since these compounds can readily donate hydrogen atoms to the radical. On the other hand, Tang et al. [33] indicated the lack of antiradical activity of methanol extract from parsley. The highest antiradical activity was determined for ethanol extract from green tea. Interestingly, the simulated digestion caused a decrease in antiradical activity in all the raw materials tested (with the exception of mustard). These types of impacts are documented in the literature [34].

### 3.3. Thyroid Peroxidase Inhibitory Potential

**Figure 1:**
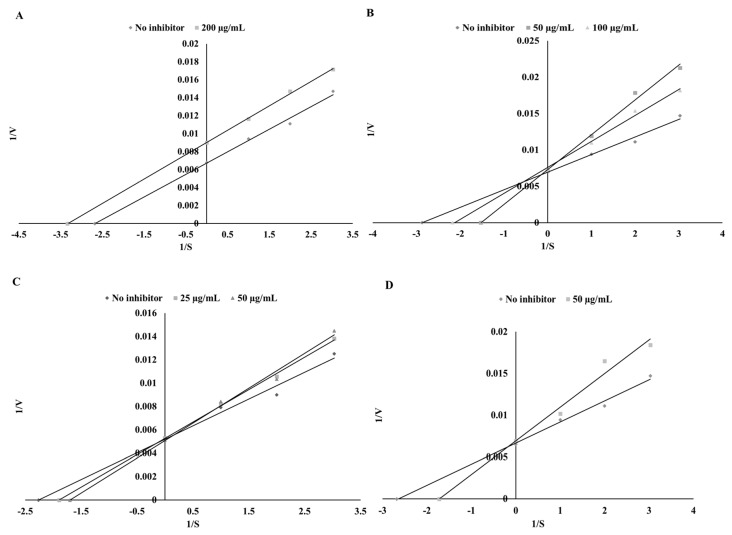
Mode of TPO inhibition by pure polyphenols: apigenin (**A**), catechin (**B**), kaempferol (**C**), and sinapinic acid (**D**).

The next step of our investigation was to determine the inhibitory potential of the tested polyphenols and prepare ethanol and digested extracts with anti-TPO activity. As expected, the pure chemical solutions demonstrated high TPO inhibitory activity. Pure substances showed a dose-independent inhibitory effect, thus effective concentrations (EFC_50_) inhibiting TPO in 50%, of pure polyphenolic substances were used in the study. The highest TPO-inhibitory activity was found for sinapinic acid and catechin, whereas the lowest was for apigenin (EFC_50_ = 25.42, 29.76, and 116.28 µg/mL, respectively). The chosen polyphenols showed a different mode of TPO inhibition (Table 3). Catechin, sinapinic acid, and kaempferol acted as competitive inhibitors, while apigenin demonstrated an uncompetitive mode of inhibitory action (Figure 1).

**Figure 2:**
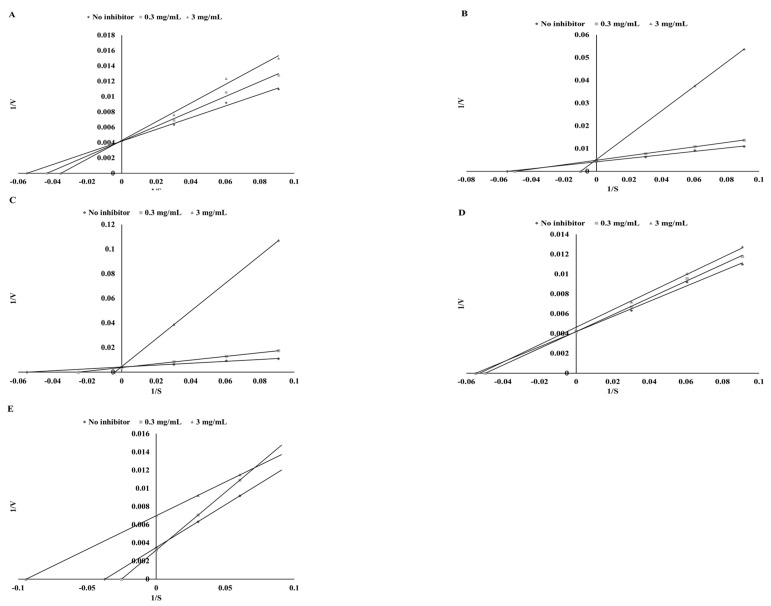
Mode of TPO inhibition by ethanol extracts from parsley (**A**), green coffee (**B**), green tea (**C**), cumin (**D**), and mustard (**E**).

**Figure 3:**
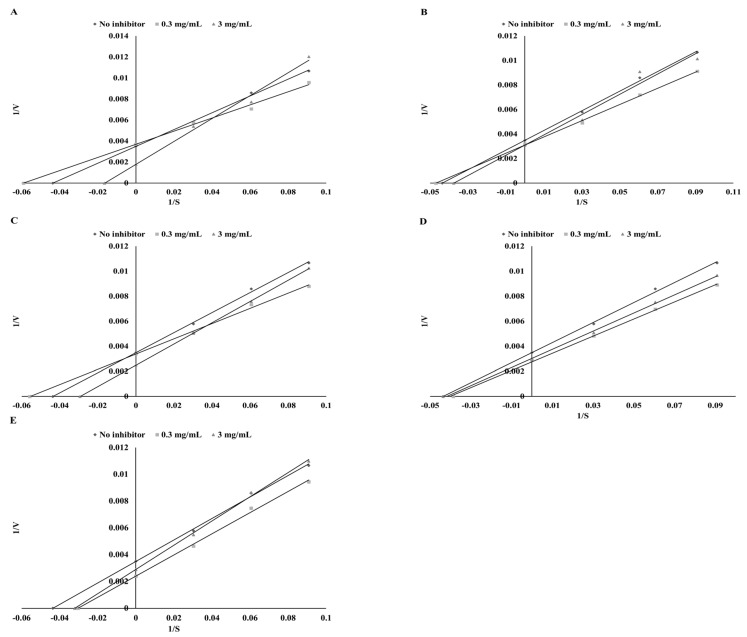
Mode of TPO influence by digested extracts of parsley (**A**), green coffee (**B**), green tea (**C**), cumin (**D**), and mustard (**E**).

Based on preliminary studies two concentrations of the extracts were chosen, i.e., 0.3 mg DW/mL and 3 mg DW/mL. Depending on the content of each polyphenol in the tested extracts, some interrelations were observed. As a rich source of catechin, green tea showed a competitive mode of inhibition in the case of the ethanol extracts and activation in the case of the digested extracts (Figure 2 and Figure 3). The same result was observed for cumin and kaempferol (Figure 1) as well as for green coffee and catechin (Figure 2 and Figure 3). In the case of sinapinic acid, a competitive mode of inhibition was observed for the pure substance and for mustard ethanol extract (at a concentration of 0.3 mg DW/mL). For higher concentrations, a competitive mode of inhibition was found (Figure 3). In the case of digested extract from mustard, slight activation was observed for concentrations of 0.3 mg DW/mL (Figure 3). Different modes of inhibition were observed, depending on the concentration of the extract used. Apigenin showed an uncompetitive mode of inhibition, whereas the ethanol extract of parsley (a rich source of apigenin) exhibited a competitive mode of inhibition (Figure 2). The digested parsley extract showed activation at the lower concentration and mixed type of inhibition for the higher concentration. The possible cause of these differences may be the influence of the food matrix. As shown, simulated digestion can strongly affect the final mode of action against the TPO enzyme. While the ethanol extracts from the tested plants inhibited the activity of TPO, conditions occurring during in vitro digestion resulted in the loss of the ability to inhibit this enzyme. Most importantly, in all cases, the ability to activate the TPO enzyme was observed. The highest ability to activate TPO was found for potentially bioaccessible compounds from mustard (24.66%) and cumin (19.85%) (Table 4). Similar dependencies were reported by Gawlik-Dziki et al. [35] during studies on the effect of plant extracts on LOX activity.

In our investigation, unexpected results were obtained using classic Lineweaver–Burk analysis. As shown in the graph below (Figure 3), the line without an inhibitor runs higher than that with the inhibitor, which indicates activation.

### 3.4. Lipoxigenase Inhibitory Potential

**Figure 4:**
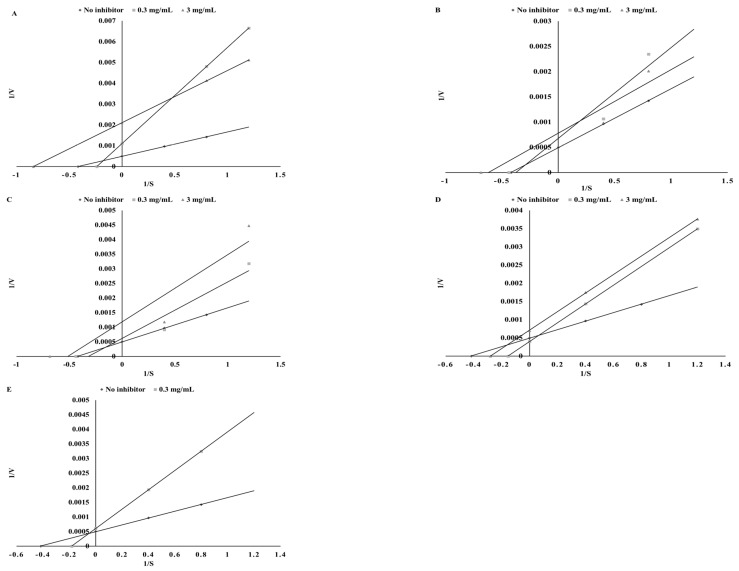
Mode of LOX inhibition by ethanol extracts from parsley (**A**), green coffee (**B**), green tea (**C**), cumin (**D**), and mustard (**E**).

**Figure 5:**
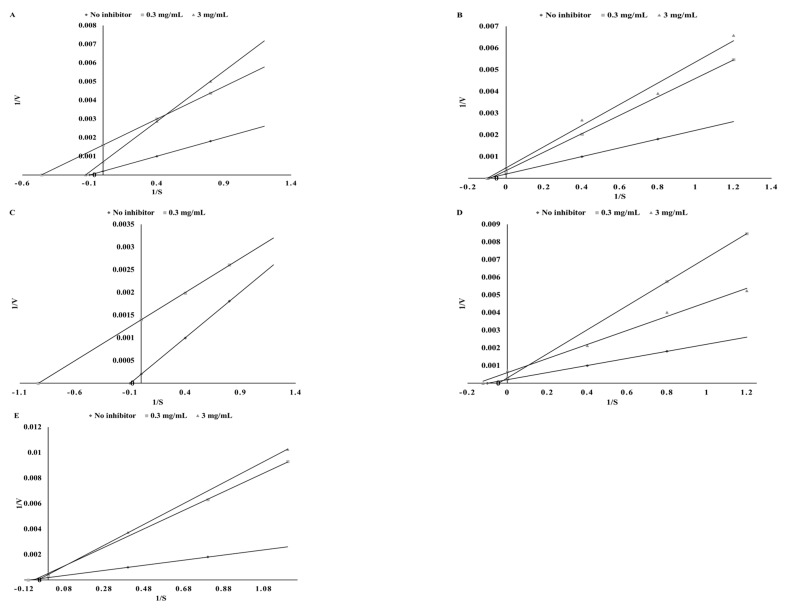
Mode of LOX inhibition by digested extracts from parsley (**A**), green coffee (**B**), green tea (**C**), cumin (**D**), and mustard (**E**).

Another very interesting question was whether there was some dependency among the modes of action of TPO-effective plant extracts with LOX. As presented in Table 5, the highest LOX inhibitory potential was found for green tea and green coffee ethanol extracts (IC_50_ = 13.74 and 15.96 mg DW/mL), whereas the lowest was in the case of mustard extract (29.01 mg DW/mL). As presented in Table 5 and Figure 4, a mixed type of LOX inhibition was obtained in most of the ethanol extracts. Only in the case of mustard ethanol extract was a competitive mode of inhibition observed. After simulated digestion, the mode of LOX inhibition changed in most of the extracts except for mustard (Figure 5).

### 3.5. Interaction Assay

Many studies have been undertaken to explore the influence of particular drug components on TPO activity and interactions among these components. The data on the influence of plant extracts on in vitro TPO activity are sparse. As described above, there are literature data confirming that polyphenolic compounds have an impact on enzymatic activity, but there are very limited data about the influence of polyphenolic compounds on TPO activity. It is known that polyphenolics contained in food sources appear in more complex combinations. Additionally, medicines with more than one active substance are more effective [36].

As shown in Figure 3, potentially bioaccessible compounds from tested plants activate TPO. This is very important especially for people suffering from Hashimoto’s disease. However, the intake of the amount giving a significant effect is in practice very difficult, which is why the next step was to determine the type of interaction among the tested extracts. Isobolographic analysis is a method used for identification of interactions among active compounds. This method is independent of the mechanism of activity; it is a useful tool for determination of interactions between the components of two-component mixtures, as well as those composed of plant extracts being mixtures of many active compounds [15].

**Figure 6:**
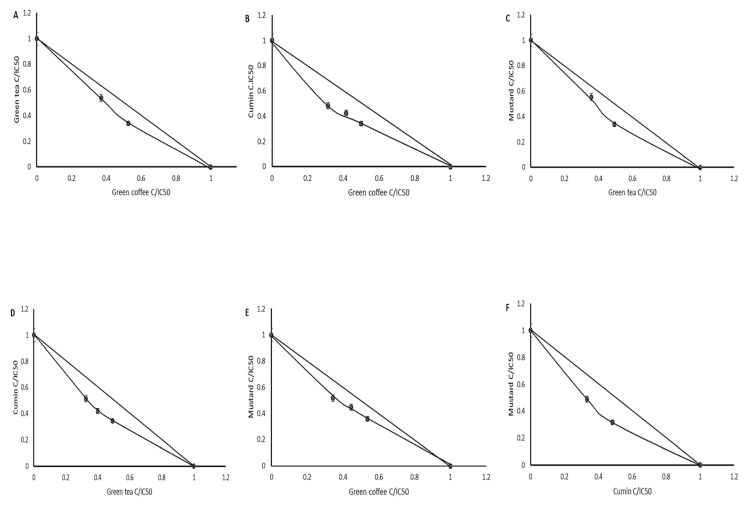
Dose-normalized isobolograms for digested extracts from green tea and green coffee (**A**), cumin and green coffee (**B**), mustard and green tea (**C**), cumin and green tea (**D**), mustard and green coffee(**E**), mustard and cumin (**F**) affecting TPO activity.

As shown in the graphs (Figure 6), the digested extract acted synergistically in all mixtures.

Isobolographic analysis is mainly used for characterization of pharmaceuticals. There are not much data about the influence of polyphenolic combinations on TPO activity. As mentioned above, scientists pay attention mainly for the interactions among pharmaceuticals, but there are some studies focused also on the interactions between food additives or food sources. These data are relatively new. Studies provided by Lau et al. [37] show the synergistic type of interaction among popular food additives such as aspartame, Quinoline Yellow, and between Brilliant Blue and L-glutamic acid. Synergism means that the occurrence of both substances in consumed food has a higher impact on humans’ health than the occurrence of each substance separately. Used combinations of additives showed higher neurotoxicity by reducing the length of neurite outgrowth. Knowledge about the interactions of substances different than pharmaceuticals is still scarce. The lack of information generates a need for these kinds of studies.

The influence of pure phenolic substances on TPO activity has been shown by Divi and Doerge [38]. There are some studies showing the influence of phytochemical compounds on the activity of other enzymes, e.g., lipoxygenase. Studies conducted by Durak et al. [39] demonstrated inhibitory activity of pure and dietary polyphenols on LOX activity and interactions among these inhibitors. The biological advantages of polyphenols included in plant sources of food appear useful for the development of bioactive functional food. Potent TPO effectors (inhibitors and activators) and potent inhibitors of LOX could be dedicated for subjects with some health diseases, e.g., hyperthyroidism and hypothyroidism. The occurrence of these types of diseases is associated with inflammation caused by the activity of prooxidative enzymes. Selection of foods with documented desirable features may provide dual benefits to human health. The raw material and the proposed functional combinations may be helpful in dietary therapy and prevention of thyroid dysfunctions correlated with an increase in the activity of LOX. This study highlights the need for the testing of interactions between active ingredients of designed functional products.

## 4. Conclusions

Green coffee, green tea, cumin, and mustard contain potentially bioaccessible TPO activators that also act as effective LOX inhibitors, which may point to their potentially health-promoting effects for people suffering from Hashimoto’s disease. It should be emphasized, however, that these are preliminary tests carried out in model systems and require further verification. Additionally, the results presented in this paper indicate a number of factors that complicate the biochemical studies that have been used for years. First is the dose-independent mode of action, which makes it impossible to set IC_50_ values. In addition, in many cases, the mechanism of inhibition varies with the concentration of the compound/extract.

In the case of activators, it is also difficult to determine classic kinetic parameters based on the classic Lineweaver–Burk analysis. The obtained results also indicate clearly that the prediction of biological activity of plant extracts based only on their chemical composition may lead to erroneous inference, because the key role in their case is played by changes occurring during digestion and/or interactions with other components of the food matrix.

## Figures and Tables

**Table 1:** Concentration of chosen polyphenols in ethanol extracts (*n* = 9).

Plant Source	Polyphenol	Concentration (mg/g DW)
Green coffee	Catechin	47.7 ± 0.23 ^a^
Green tea	Catechin	11.5 ± 0.58 ^b^
Parsley	Apigenin	0.69 ± 0.034
Cumin	Kaempferol	0.09 ± 0.004
Mustard	Sinapinic acid	1.25 ± 0.627

The values are expressed as the mean ± SD; means with different letter superscripts (^a^,^b^) are significantly different (α = 0.05).

**Table 2:** Comparison of ABTS radical scavenging ability (expressed as IC_50_ values) of pure chemicals, ethanol plant extracts (EEs), and digested plant extracts (DEs) (*n* = 9).

**Pure chemical standards**	**IC_50_ (mg/mL)**	
**Sinapinic acid**	0.084 ± 0. 001 ^a^	
Apigenin	0.406 ± 0.003 ^c^	
Catechin	0.078 ± 0.008 ^a^	
Kaempferol	0.337 ± 0.002 ^b^	
**Plant extracts**	**IC_50_ (mg DW/mL)**	
**Plant**	**EE**	**DE**
Parsley	3.47 ± 0.17 ^e^	1.09 ± 0.05 ^c^
Green coffee	0.78 ± 0.04 ^d^	0.87 ± 0.04 ^b^
Green tea	0.06 ± 0.0003 ^a^	1.33 ± 0.06 ^d^
Cumin	0.32 ± 0.01 ^b^	6.52 ± 0.32 ^e^
Mustard	0.41 ± 0.02 ^c^	0.42 ± 0.02 ^a^

Values are expressed as the mean ± SD; means with different letter superscripts (^a^–^e^) in the columns are significantly different (α = 0.05). ABTS -(2,2′-azinobis-(3-ethylbenzothiazoline-6-sulfonic acid, IC_50_—The half maximal inhibitory concentration.

**Table 3:** The EFC_50_ (effective concentration), Ki, and V_max_ values and mode of thyroid peroxidase inhibition of catechin, kaempferol, sinapinic acid, and apigenin solutions (*n* = 9).

Substance	Mode of Inhibition	EFC_50_ (µg/mL)	Ki (µg/mL)	V_max_ (ΔAU/min)
Sinapinic acid	Competitive	25.42 ± 1.13 ^a^	45.92 ± 3.22 ^b^	149.25 ± 6.26 ^a^
Apigenin	Uncompetitive	116.28 ± 5.38 ^d^	338.73 ± 12.31 ^d^	149.25 ± 5.12 ^a^
Catechin	Competitive	29.76 ± 2.05 ^b^	33.42 ± 1.35 ^a^	144.93 ± 4.33 ^a^
Kaempferol	Competitive	61.68 ± 3.12 ^c^	186.95 ± 6.89 ^c^	192.31 ± 8.34 ^b^

The values are expressed as the mean ± SD; means with different letter superscripts (^a^–^d^) in the columns are significantly different (α = 0.05).

**Table 4:** IC_50_ value, Ki value, V_max_ value and impact on TPO of ethanol and digested extracts from parsley, cumin, green tea, green coffee, and mustard (*n* = 9).

Plant	Ethanol Extracts				Extracts after Digestion in Vitro	
	Mode of Inhibition	IC_50_ (mg DW/mL)	Ki (mg DW/mL)	V_max_ (Δ AU/min)	Action	% of Activation
Parsley	competitive	103.45 ± 3.12 ^ab^	387.42 ± 11.53 ^c^	238.1	activation	0.39 ± 0.06 ^a^
Green coffee	competitive	185.01 ± 6.32 ^d^	1971.0 ± 53.5 ^d^	238.1	activation	17.73 ± 1.12 ^c^
Green tea	competitive	126.26 ± 4.15 ^c^	107.12 ± 3.15 ^a^	238.1	activation	14.07 ± 0.92 ^b^
Cumin	competitive	100.00 ± 3.21 ^a^	1891.5 ± 42.6 ^d^	238.1	activation	19.85 ± 1.30 ^c^
Mustard	competitive	106.13 ± 4.15 ^b^	218.4 ± 5.39 ^b^	285.7	activation	24.66 ± 1.42 ^d^

The values are expressed as the mean ± SD; means with different letter superscripts (^a^–^d^) in the columns are significantly different (α = 0.05).

**Table 5:** IC_50_ value, Ki value, Vmax value, and mode of LOX inhibition by ethanol and digested extracts from parsley, cumin, green tea, green coffee, and mustard (*n* = 9).

Plant	Ethanol Extracts			Extracts after Digestion in Vitro		
	Mode of Inhibition	IC_50_ (mg DW/mL)	Ki (mg DW/mL)	Mode of Inhibition	IC_50_ (mg DW/mL)	Ki (mg DW/mL)
Parsley	mixed	24.39 ± 1.18^b^	32.85 ± 1.43^d^	uncompetitive	45.05 ± 1.98^c^	6.44 ± 0.53^b^
Green coffee	mixed	15.96 ± 1.12^a^	2.82 ± 0.16^a^	noncompetitive	30.43 ± 1.52^a^	30.43 ± 1.16^d^
Green tea	mixed	13.74 ± 0.92^a^	2.84 ± 0.21^a^	uncompetitive	29.73 ± 1.12^a^	4.96 ± 0.19^a^
Cumin	mixed	21.54 ± 1.23^b^	12.61 ± 0.96^b^	competitive	34.13 ± 0.85^b^	26.94 ± 0.97^c^
Mustard	competitive	29.01 ± 1.41^c^	22.46 ± 1.18^c^	competitive	32.01 ± 1.02^ab^	72.74 ± 2.16^e^

The values are expressed as the mean ± SD; means with different letter superscripts (^a^–^e^) in the columns are significantly different (α = 0.05).
